# Socio-Economic Position and Suicidal Ideation in Men

**DOI:** 10.3390/ijerph14040365

**Published:** 2017-03-31

**Authors:** Jane Pirkis, Dianne Currier, Peter Butterworth, Allison Milner, Anne Kavanagh, Holly Tibble, Jo Robinson, Matthew J. Spittal

**Affiliations:** 1 Centre for Mental Health, Melbourne School of Population and Global Health, University of Melbourne, Melbourne 3010, Australia; peter.butterworth@unimelb.edu.au (P.B.); alison.milner@unimelb.edu.au (A.M.); a.kavanagh@unimelb.edu.au (A.K.); holly.tibble@unimelb.edu.au (H.T.); m.spittal@unimelb.edu.au (M.J.S.); 2 Centre for Epidemiology and Biostatistics, Melbourne School of Population and Global Health, University of Melbourne, Melbourne 3010, Australia; dianne.currier@unimelb.edu.au; 3 Centre for Health Equity, Melbourne School of Population and Global Health, University of Melbourne, Melbourne 3010, Australia; jo.robinson@orygen.org.au

**Keywords:** socio-economic position, suicidal ideation, disadvantage

## Abstract

People in low socio-economic positions are over-represented in suicide statistics and are at heightened risk for non-fatal suicidal thoughts and behaviours. Few studies have tried to tease out the relationship between individual-level and area-level socio-economic position, however. We used data from *Ten to Men* (the Australian Longitudinal Study on Male Health) to investigate the relationship between individual-level and area-level socio-economic position and suicidal thinking in 12,090 men. We used a measure of unemployment/employment and occupational skill level as our individual-level indicator of socio-economic position. We used the Index of Relative Socio-Economic Disadvantage (a composite multidimensional construct created by the Australian Bureau of Statistics that combines information from a range of area-level variables, including the prevalence of unemployment and employment in low skilled occupations) as our area-level indicator. We assessed suicidal thinking using the Patient Health Questionnaire (PHQ-9). We found that even after controlling for common predictors of suicidal thinking; low individual-level and area-level socio-economic position heightened risk. Individual-level socio-economic position appeared to exert the greater influence of the two; however. There is an onus on policy makers and planners from within and outside the mental health sector to take individual- and area-level socio-economic position into account when they are developing strategic initiatives.

## 1. Introduction

Inequities in health are of major concern to policy-makers and practitioners across the board. Health inequities can be thought of as the subset of inequalities—or differences—in the health of individuals and groups that are value-based and underpinned by injustice, unfairness or avoidability [1]. A major driver of health inequities is socio-economic position. Socio-economic position is a complex construct which relates to the differential opportunities individuals, households and communities have to access the resources that enable good health. Different indicators of socio-economic position capture different components of this construct at an individual level. For example, education best captures human capital in terms of the capacity of individuals to access and understand information. Education also shapes people’s job opportunities and future income. Occupation has traditionally been thought of as a measure of social status depending on the prestige of the particular occupation. Occupation also captures exposure to physical, and potentially psychological, workplace hazards. Occupation is a strong determinant of health status, particularly for men. Employment status is a major determinant of health, reflecting social status as well as access to material and psychosocial resources, while income level reflects the material resources of individuals and households [2]. It is also possible to measure area-level indicators of socio-economic position, using indicators like the percentage of an area’s population with low levels of education or on low incomes. Area-level measures encapsulate the social and economic resources within areas. Health outcomes show different patterns according to the indicator of socio-economic position used.

In the suicide prevention field, a number of studies have explored inequities in suicide and, to a lesser extent, non-fatal suicidal outcomes (i.e., suicide attempts and suicidal ideation). As in other areas of health, socio-economic position, as assessed by different indicators, has been a major focus. Platt provides an excellent overview of these studies in his chapter in the recently published *International Handbook of Suicide Prevention* [3]. Individual-level studies consistently show over-representation in suicide statistics and heightened risk for non-fatal suicidal behaviour in individuals who are unemployed [4,5,6], work in unskilled jobs or jobs with poor psychosocial working conditions [7,8,9], have insecure housing [10,11] and relatively low levels of educational attainment [10,12,13]. Area-level studies produce less consistent findings, with some studies showing that relatively deprived areas (as assessed by average income, for example) have higher suicide or suicide attempt rates than more affluent areas, and others finding no such relationship [3,12,14].

Understanding the different contributions and potential interactions between individual- and area-level socio-economic position is important for the design of suicide prevention programs. Relatively few studies have been conducted, however, that consider the impact of individual- and area-level socio-economic position simultaneously, at least in general adult populations, although some studies have been done [3,15,16,17]. Two plausible hypotheses have been advanced, taken from the broader literature on health inequities. One is that area-level socio-economic position may compound individual-level socio-economic position, with the two acting in an additive fashion and maximising the risk. Under this scenario, those in the lowest socio-economic positions in areas of the greatest disadvantage would be at the greatest risk. The second hypothesis is that there may be a more complex interaction between individual-level and area-level socio-economic position, such that the effect of individual-level socio-economic position might be more pronounced in areas of lower socio-economic position (i.e., greater advantage). This might play out in circumstances where individuals who are in low status occupations are at greater risk if they perceive themselves to be surrounded by peers in higher status occupations than if they perceive themselves to reflect the norm for their area. Similarly, unemployed individuals may be more marginalised if they are living in more affluent areas, or, conversely, they may benefit from the additional resources available to them [18].

We had the opportunity to contribute further to knowledge in this area by investigating the relationship between individual-level and area-level socio-economic position and suicidal ideation in a nationally representative cohort of men because of our involvement in *Ten to Men*, the Australian Longitudinal Study on Male Health [19,20]. We used a composite measure of unemployment/employment and occupational skill level. Our aim was to determine whether each of these factors demonstrated an independent association with suicidal ideation and, if so, how the two might work in combination.

Our focus on men was deliberate. Three quarters of all suicides in Australia are by males [21]. Occupation is a well-described measure of men’s social status and social status has been strongly linked to suicide. Unemployed men—who arguably have the lowest social status—have an extremely heightened risk of suicide [22]. Examining the impact of these indicators of socio-economic position for men at the individual- and area-level, and focusing on suicidal ideation rather than suicide, could provide useful information to guide future suicide prevention activities.

## 2. Materials and Methods

The approach we used in *Ten to Men* has been described in detail elsewhere [19,20]. Here we provide a brief overview of the sampling and recruitment strategy and the data collection process. We also describe the variables we used in the current analysis, and the analysis strategy itself.

Ten to Men was approved by the University of Melbourne Human Research Ethics Committee (HREC 1237897 and HREC 1237376). Ten to Men data are available at the Australian Data Archive (DOI:10.4225/87/587ebdbc851b1.)

### 2.1. Sampling and Recruitment

In *Ten to Men*, we recruited a cohort of 15,988 males aged 10–55 in 2013/14 using a stratified, multi-stage, cluster random sampling strategy. We approached the full complement of households (*n* = 104,884) in 622 randomly-selected statistical areas (SA1s) from across Australia. SA1s are the smallest unit for which the Australian Bureau of Statistics will release census data, and have populations ranging from about 200 to 800. In total, we made contact with 81,400 (78%) of these households and identified 33,724 (32%) as having at least one male resident of the appropriate age. Ultimately, we identified 45,510 ‘in scope’ males in these households, so the 15,988 who agreed to be part of the cohort and returned usable data represented a response fraction of 35%. For the purposes of the current study, we focused on the 13,884 18–55 year-old men in the cohort.

### 2.2. Data Collection

At recruitment, we collected baseline (Wave 1) data from the 18–55 year-old via self-complete questionnaires. These men were asked questions that covered five broad domains (physical health, mental health and wellbeing, health behaviours, social determinants of health, and health service utilisation and health knowledge).

### 2.3. Primary Outcome: Suicidal Ideation

Our primary outcome was a single item taken from the Patient Health Questionnaire (PHQ-9) [23]. This question asks “Over the past two weeks, how often have you been bothered by thoughts that you would be better off dead, or of hurting yourself in some way?” Responses to this question are: (a) not at all; (b) several days; (c) more than half the days; and (d) nearly every day. Scores are assigned from 0 to 3, respectively. For the purposes of our analysis, we considered those who scored 1 (i.e., were bothered by these thoughts for several days or more) or more to be experiencing suicidal ideation. It is worth noting here that this single PHQ-9 item has been shown to have good specificity and reasonable sensitivity when compared to a structured clinical interview [24], and was recently shown to predict suicide risk in a large-scale register-based study of (predominantly male) Veterans Administration (VA) patients in the United States [25].

### 2.4. Exposure Variables: Individual-Level and Area-Level Socio-Economic Position

We measured individual-level socio-economic position by considering responses to questions on employment status and occupation type. The employment status question asked: “Are you currently: (a) employed/working for profit or pay; (b) unemployed and looking for work; or (c) neither working nor looking for work?” Those who indicated they were employed were asked: “What is your current occupation (in your main job)?” and given the opportunity to provide a free text response. These responses were then coded according to the Australian and New Zealand Standard Classification of Occupations (ANZSCO) by occupational skill level: (a) low (sales, machinery workers, and labourers); (b) medium (technical and trade workers, community and personal service workers, and clerical and admin workers); and (c) high (managers and professionals) [26]. We created a single variable that was designed to represent a gradient of individual-level socio-economic status by combining these responses into the following categories: (a) unemployed; (b) working in low skilled occupation; (c) working in medium skilled occupation; and (d) working in high skilled occupation.

We measured area-level socio-economic position using the Index of Relative Socio-economic Disadvantage (IRSD) [27]. The IRSD is one of four composite indexes in the Socio-Economic Indexes for Areas (SEIFA), created by the Australian Bureau of Statistics to summarise the social and economic conditions of areas (in our case, SA1s) using census information. The IRSD focuses on disadvantage. It is a multidimensional construct that reflects the disadvantage of individuals, families and dwellings within areas. It is constructed using data reduction techniques (principal component analysis) to combine information from a range of variables assessing the prevalence within areas of unemployment, employment in low skilled occupations, low income, low levels of educational attainment, overcrowding, and other social correlates of disadvantage. A low IRSD score is indicative of relatively greater disadvantage, and a high IRSD score denotes relatively lesser disadvantage. The Australian Bureau of Statistics distributes SA1s in Australia into quintiles based on the IRSD; SA1s in Quintile 1 are the most disadvantaged and SA1s in Quintile 5 are the least disadvantaged. We used these values for our study.

It is worth noting here that we deliberately chose our individual-level indicator of socio-economic position on the basis of it having a degree of consistency with our area-level indicator. As noted, unemployment and employment in low skilled occupations feature as measures that are used to build up the composite IRSD index.

### 2.5. Covariates

We also controlled for the influence of a number of socio-demographic and clinical covariates, largely selecting these on the basis that they have been shown in previous studies to be associated with suicidal ideation in males. These were age, marital status, region of residence, level of social support, alcohol use and depression. The way in which each of these was operationalised is described below:

- Age was classified into four broad groupings: (a) 18–29; (b) 30–39; (c) 40–49; and (d) 50–55.
- Marital status was dichotomised: (a) married/de facto; and (b) never married/widowed/divorced/separated.
- Region of residence was defined using the Remoteness Area classification of the Australian Statistical Geography Standard (ASGS), which splits SA1s into: (a) major cities; (b) inner regional areas; and (c) outer regional areas [28].
- Level of social support was taken from the emotional/informational support sub-scale of the Medical Outcomes Survey Social Support Survey (MOS-SS). This asks about how often various kinds of social support are available to the respondents and generates a scaled score from 0 to 100. Higher scores denote greater social support [29].
- Alcohol use was measured by the Alcohol Use Disorders Identification Test (AUDIT). The AUDIT assess alcohol consumption, drinking behaviours and alcohol-related problems, and classifies respondents’ alcohol use as: (a) harmful/hazardous; and (b) not harmful/hazardous [30].
- Depression was ascertained by a two questions that were taken from the Australian Health Survey. The first of these asked “Has a doctor or other health professional ever told you that you had depression?” Those who answered “yes” to this were then asked “Have you been treated for or had any symptoms of depression in the past 12 months?” Those who again answered “yes” were deemed to have experienced depression in the past 12 months [31].

### 2.6. Analysis

We conducted univariate and multivariate logistic regression analyses to evaluate the strength of association between individual-level and area-level socio-economic position and suicidal ideation. In each multivariate analysis, we controlled for the alternative measure of socio-economic position and for each of the other covariates. Based on the regression model coefficients, we also calculated the probability of suicidal ideation for each grade of individual-level socio-economic position within each quintile of area-level socio-economic position.

All data were analysed using Stata (Version 13.1) [32].

## 3. Results

In order to be included in the current analysis, the men in the cohort had to have provided data that allowed us to classify them on the basis of the primary outcome (suicidal ideation) and the main exposure variables (individual-level and area-level socio-economic position). This meant that we excluded those who had missing data on the suicidal ideation variable or the individual-level socio-economic position variable; it was not possible for them to have missing data on the area-level socio-economic position variable because this variable was automatically generated on the basis of the SA1 in which they lived. We also excluded those who indicated that they were neither working nor looking for work (*n* = 829), because it was not possible to classify this group as unemployed or working in one of the three occupational skill levels.

In total, we were able to use data from 12,090 (87%) of the 13,884 18–55 year-old in the *Ten to Men* cohort for the current study. The sample included representation from men of varying ages, with 23.8% aged 18–29 years, 27.4% aged 30–39 years, 31.5% aged 40–49 years, and 17.3% aged 50–55 years.

Table 1 shows the distribution of the sample on the outcome and exposure variables. In total, 8.6% had experienced suicidal thoughts in the past two weeks. In terms of individual-level socio-economic position, 9.3% were unemployed, 23.2% were working in low skilled occupations, 32.7% were working in medium skilled occupations, and 34.7% were working in high skilled occupations. They were fairly uniformly distributed across more and less socio-economically disadvantaged areas, with around one fifth living in SA1s in each IRSD quintile (17.3% in Quintile 1, 18.6% in Quintile 2, 23.2% in Quintile 3, 21.0% in Quintile 4, and 19.9% in Quintile 5).

Table 2 gives additional detail about the two main exposure variables, individual-level and area-level socio-economic position. It shows that within each quintile of area-level disadvantage, there was a broad spread of individual-level socio-economic status. For example, 19.1% of the men living in SA1s in Quintile 1 (SA1s of greatest socio-economic disadvantage) were working in occupations of the highest skill level. Similarly, 5.9% of the men living in SA1s in Quintile 5 (SA1s of least socio-economic disadvantage) were unemployed, and a further 14.5% were working in low skilled occupations.

Table 3 shows the results of the logistic regression analyses. In the univariate analysis, individual-level and area-level socio-economic position were both predictive of suicidal ideation, with lower socio-economic status being associated with higher odds of experiencing suicidal ideation. In each case, after controlling for the alternative indicator of socio-economic position and the other covariates, both individual-level and area-level indicators of socio-economic position remained significant, although the magnitude of the area-level effect reduced substantially.

**Figure 1:**
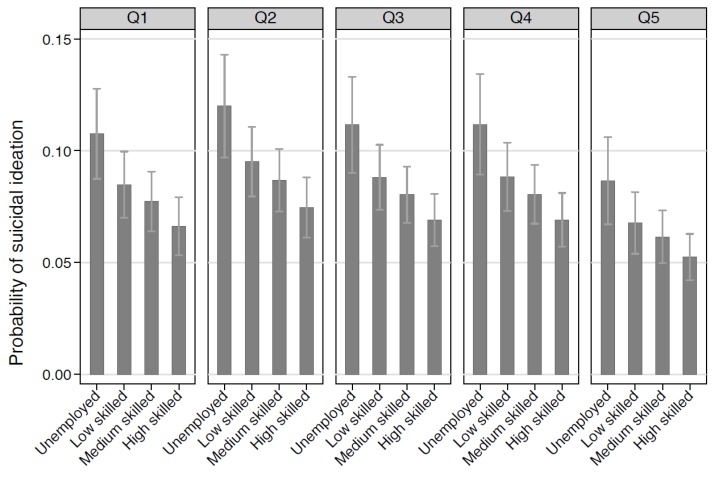
Probability of suicidal ideation by individual-level and area-level disadvantage *. * Adjusting for age, marital status, region of residence, level of social support, alcohol use and depression.

Figure 1 builds on the findings in Table 3 by describing the probability of suicidal ideation by individual-level and area-level socio-economic position. It shows that within each quintile of area-level disadvantage there is a gradient of risk, such that the probability of suicidal ideation is highest for unemployed men and lowest for men working in high skilled occupations. The picture is similar for each quintile, reinforcing the relative importance of individual-level socio-economic status.

In a final analysis, we tested for an interaction between individual-level and area-level socio-economic position, controlling for covariates. We found no evidence of such an association (*p* = 0.98).

## 4. Discussion

This study aimed to explore whether individual-level and area-level socio-economic position are associated with suicidal thinking in men, and if so, whether one compounds the other. We found that even after controlling for other common predictors of suicidal thinking, low individual-level and area-level socio-economic status heightened risk. Individual-level socio-economic position—as measured by unemployment/employment and occupational skill level—appeared to exert the greater influence of the two, however.

### 4.1. Mechanisms by Which Low Individual-Level and Area-Level Socio-Economic Status May Contribute to Suicidal Thinking

As with other areas of health, it is easy to see how low individual-level socio-economic status might contribute to suicidal thinking. We know from elsewhere in the suicide prevention literature that life stressors can sometimes act as a trigger, particularly if underlying or longer-standing risk factors are already present [33]. Some of the ways in which individual-level socio-economic disadvantage manifests itself in relation to employment and occupational skill level—the indicators we used here—certainly qualify as life stressors. For example, those who are unemployed or working in low skilled occupations may experience financial strain, powerlessness, frustration and disrespect [34,35], all of which may act as potential tipping points.

Similarly, it is possible to see how the role of area-level socio-economic status may play out, with, for example, poorer areas having less in the way of infrastructure and services that may help to prevent individuals getting to the point where they view suicide as an option. For instance, areas of relatively greater socio-economic disadvantage may be less well served by mental health services. Perhaps even more importantly, other social, economic, cultural and physical features of these areas may also have a negative impact on the sense of wellbeing of their residents [36]. These features might include, for example, high levels of inter-generational poverty, community norms that foster stigma and discrimination, and built environments with poor amenity that promote social isolation.

### 4.2. Implications for Policy and Practice

Around the world, national and local suicide prevention strategies have placed insufficient emphasis on socio-economic position as a risk factor for suicide and non-fatal suicidal behaviours and thoughts. Platt argues for a redirection of policy focus in this regard, noting that care should be taken about the approach. In particular, he notes that governments should weigh up the relative merits of targeting individual-level or area-level disadvantage. His view is that targeting individuals within areas will yield the greatest benefits [3]. Our findings lend support to this assertion, which is consistent with the thrust of the recent World Health Organization report *Preventing Suicide: A Global Imperative* [37].

Area-level suicide prevention efforts are gaining traction because of the popularity of systems-based approaches delivered through initiatives like the European Alliance against Depression and the OSPI-Europe project [38]. In Australia, we have one such initiative, known as Lifespan (http://www.lifespan.org.au/). These initiatives take suicide prevention strategies for which there is a reasonable evidence base and deliver them to whole communities in a co-ordinated, integrated manner. The idea is that the whole is greater than the sum of the parts, and that by using a systematic approach—as opposed to a more disjointed, piecemeal one—greater benefits will ensue. Typically, areas that are selected for these systems-based initiatives are those which have high rates of suicide and/or suicide attempts; often, but not always, these will also be socially disadvantaged areas.

The suicide prevention initiatives in these area-level initiatives usually cover the full gamut of universal interventions (target whole populations, with the aim of favourably shifting risk factors across the entire population), selective interventions (interventions target those who are not yet manifesting suicidal thoughts or behaviours, but exhibit risk factors that predispose them to do so in the future) and indicated interventions (which are designed for those who are already beginning to display suicidal thoughts or behaviours, usually identified through screening programs or by clinical presentation). In the current context, there would seem to be opportunities for selective interventions that target those whose individual-level socio-economic position puts them at risk, doing so within area-level initiatives. This would mean that individuals with low socio-economic status would be targeted in socio-economically disadvantaged areas, but also, potentially, in better-off areas.

The way in which these policy and practice implications might be operationalised for men requires some thought. In areas of low socio-economic status, consideration might be given to how to increase the availability of mental health and suicide prevention services in general, and how to ensure that they are attractive and accessible to men in particular. Of course, efforts beyond the health and mental health systems will also be required in these areas. These might include urban regeneration or economic renewal programs (e.g., in areas where there have been widespread downturns in male-dominated industries like construction, car manufacturing or mining). They might also include efforts to improve the conditions in which men in these industries are working [39].

Unemployed men and men in low status occupations in these or other areas might be targeted through tailored programs. Again, some of these programs might be delivered through the mental health system; we know that men respond differently from women to some forms of psychotherapy, so gender-specific approaches may be required. Other programs may be broader in focus and aimed at addressing disadvantage itself. These might include training and education programs designed to upskill unemployed men or men working in relatively low skilled occupations.

### 4.3. Study Limitations

Our study had several limitations which must be acknowledged here. Firstly, we used a measure of individual-level socio-economic position that comprised a composite of unemployment/employment and occupational skill level. As noted above, there are different ways of conceptualising socio-economic position, and our indicator is just one of these. Some might argue that our individual-level indicator and our area-level indicator were not sufficiently well aligned, and that they capture different components of the construct of socio-economic position. It is worth noting here, however, that unemployment/employment and occupation skill level are both used by the Australian Bureau of Statistics to build the aggregate area-level IRSD, creating some level of comparability.

Secondly, and on a related point, we relied on a single measure of individual-level socio-economic position, rather than exploring a fuller set of individual-level indicators. This means that we cannot comment on the extent to which unemployment/employment and occupational skill level act as a proxy for or behave differently from other individual-level indicators of socio-economic position. Having said that, it is worth noting that we conducted a sensitivity analysis (not reported here) which used a measure of financial stress as the individual-level indicator of socio-economic position, and that this analysis yielded similar results to our primary analysis.

Thirdly, we used data from a single wave of *Ten to Men* and therefore were only able to conduct cross-sectional analyses, which limits the extent to which we can ascribe causality to the observed associations between individual-level and area-level disadvantage and suicidal ideation. We have now completed our second wave of data collection and are poised to conduct our first set of longitudinal analyses.

Fourthly, there may have been issues in terms of our covariates. As noted, we chose these because they have consistently been shown to be predictors of suicidal ideation. Some may also have had a relationship with the exposure (i.e., our unemployment/employment and occupational skill level variable), which raises questions about whether our selected covariates acted as confounders or mediators. There is an argument that we may have over-controlled for some of those which acted as mediators, thereby underestimating the effect of unemployment and relatively lower skilled occupations on suicidal ideation.

Finally, there are a range of other limitations that relate to *Ten to Men* itself. These include a reliance on self-report, and the fact that the sampling strategy meant that males in remote areas and/or who were not proficient in English were under-represented.

## 5. Conclusions

This study provides new insights into the association between individual-level and area-level indicators of socio-economic position and suicidal thinking in men, albeit on the basis of a very particular indicator of individual-level socio-economic position (a composite indicator based on unemployment/employment and occupational skill level). Previous studies in this area have tended to focus on suicide as an outcome; relatively few have considered suicide attempts, and fewer still have examined suicidal ideation. This is important because suicidal thoughts and behaviours are often conceptualised as occurring on a continuum, which means that intervening with those who are thinking about suicide may mean that subsequent non-fatal, and fatal, suicidal acts can be averted. Our study showed that individual- and area-level disadvantage both place men at risk for suicidal ideation, with the former exerting a particularly strong influence. Further work is required to determine whether our results hold up using different methods (e.g., hierarchical modelling techniques). Assuming they do, there is an onus on policy makers and planners from within and outside the mental health sector to take this into account when they are developing strategic initiatives.

## Figures and Tables

**Table 1:** Distribution of the sample (*n* = 12,090) on outcome and exposure variables.

Sample Characteristics	Value	
Suicidal ideation (past 2 weeks)—*n* (%)		
Yes	1036	(8.6%)
No	11,054	(91.4%)
Individual-level disadvantage—*n* (%)		
Unemployed	1123	(9.3%)
Low skilled occupations	2810	(23.2%)
Medium skilled occupations	3956	(32.7%)
High skilled occupations	4201	(34.8%)
Area-level disadvantage—*n* (%)		
Q1 (Most disadvantaged)	2094	(17.3%)
Q2	2245	(18.6%)
Q3	2803	(23.2%)
Q4	2541	(21.0%)
Q5 (Least disadvantaged)	2407	(19.9%)

**Table 2:** Individual-level and area-level disadvantage.

Area Disadvantage Quintile	Unemployed *n* (%)	Low Skilled Occupations *n* (%)	Medium Skilled Occupations *n* (%)	High Skilled Occupations *n* (%)	Total *n* (%)
Q1 (Most disadvantaged)	368 (17.6%)	651 (31.1%)	674 (32.2%)	401 (19.1%)	2094 (100%)
Q2	236 (10.5%)	634 (28.2%)	785 (35.0%)	590 (26.3%)	2245 (100%)
Q3	227 (8.1%)	656 (23.4%)	883 (31.5%)	1037 (37.0%)	2803 (100%)
Q4	149 (5.9%)	521 (20.5%)	853 (33.6%)	1018 (40.1%)	2541 (100%)
Q5 (Least disadvantaged)	143 (5.9%)	348 (14.5%)	761 (31.6%)	1155 (48.0%)	2407 (100%)
Total	1123 (9.3%)	2810 (23.2%)	3956 (32.7%)	4201 (34.7%)	12,090 (100%)

**Table 3:** Univariate and multivariate predictors of suicidal ideation (past 2 weeks).

Predictors	Univariate Models		Multivariate Model	
	Odds Ratio (95% CI)	*p*-Value	Odds Ratio (95% CI)	*p*-Value
**Individual-level disadvantage**		<0.001		<0.001
Unemployed	4.19 (3.44–5.10)		1.83 (1.40–2.37)	
Low skilled occupation	1.79 (1.50–2.15)		1.35 (1.09–1.68)	
Medium skilled occupation	1.40 (1.18–1.67)		1.21 (0.98–1.48)	
High skilled occupation	1.00		1.00	
**SEIFA**		<0.001		0.030
Q1	2.07 (1.67–2.58)		1.32 (1.00–1.73)	
Q2	1.86 (1.50–2.32)		1.52 (1.17–1.98)	
Q3	1.53 (1.23–1.90)		1.38 (1.07–1.78)	
Q4	1.43 (1.14–1.79)		1.39 (1.07–1.80)	
Q5	1.00		1.00	
**Age**		<0.001		0.044
18–29	1.00		1.00	
30–39	0.68 (0.58–0.81)		0.75 (0.60–0.94)	
40–49	0.70 (0.59–0.83)		0.80 (0.64–1.00)	
50–55	0.70 (0.58–0.86)		0.73 (0.56–0.94)	
**Marital status**		<0.001		<0.001
Never married/widowed/separated	2.36 (2.07–2.69)		1.48 (1.24–1.77)	
Married/defacto	1.00		1.00	
**Region of residence**		0.228		0.491
Major cities	1.00		1.00	
Inner regional areas	1.13 (0.97–1.32)		0.94 (0.78–1.13)	
Outer regional areas	0.98 (0.83–1.17)		0.89 (0.72–1.09)	
**Level of social support (MOSS-SS)**	0.97 (0.97–0.98)	<0.001	0.98 (0.97–0.98)	<0.001
**Alcohol use**		<0.001		<0.001
Not harmful/hazardous	1.00		1.00	
Harmful/hazardous	1.73 (1.51–1.98)		1.58 (1.35–1.84)	
**Depression**		<0.001		<0.001
No depression in past 12 months	1.00		1.00	
Depression in past 12 months	6.45 (5.60–7.44)		5.33 (4.52–6.30)	
